# A real-world disproportionality analysis of FDA adverse event reporting system (FAERS) events for denosumab

**DOI:** 10.3389/fphar.2024.1339721

**Published:** 2024-08-30

**Authors:** Yue He, Rong Zhang, Huarui Shen, Yingqi Liu

**Affiliations:** ^1^ Sichuan Provincial Ba-Yi Rehablitation Center (Sichuan Provincial Rehablitation Hospital), Chengdu, Sichuan, China; ^2^ Affiliated Rehabilitation Hospital of Chengdu University of Traditional Chinese Medicine, Chengdu, Sichuan, China; ^3^ Wenjiang Distrct People’s Hospital of Chengdu, Chengdu, Sichuan, China; ^4^ Department of Orthopaedics, The Affiliated Traditional Chinese Medicine, Hospital of Southwest Medical University, Luzhou, Sichuan, China; ^5^ Southwest University Hospital, Chongqing, China; ^6^ School of Materials and Energy, Southwest University, Chongqing, China

**Keywords:** denosumab, FAERS, adverse events, osteonecrosis of the jaw, RANKL (receptor activator for nuclear factor k B ligand)

## Abstract

**Background:**

Denosumab is authorized to treat several diseases, including cancer and bone disorders. Nevertheless, its use in clinical practice has been affected by safety concerns. The work retrospectively investigated adverse events (AEs) of denosumab to better understand toxicities.

**Methods:**

The FAERS data base data from Q1 of 2010 to Q3 of 2023 was chosen. The definition of Medical Dictionary for Regulatory Activities (MedDRA) was dependent on preferred terms (PTs) and system organ class (SOCs). Following the removal of duplicate reports, a disproportionality analysis was conducted to identify safety signals through the calculation of reporting odds ratios (ROR).

**Results:**

During the reporting period, 130611 denosumab-related cases were identified; 670 *p*Ts with a substantial disproportionality were retained. The connective and musculoskeletal tissue disorders, poisoning, injury, and procedural complications, as well as medical and surgical procedures, were among the important SOCs that satisfied the criteria. Reports at PT levels including off-label use, death, osteonecrosis of the jaw, arthralgia, and pain in extremities were determined. Severe consequences in terms of life-threatening injuries and death accounted for 841 and 19704 cases, respectively of the reported cases.

**Conclusion:**

These findings underscore the critical importance of pharmacovigilance and are consistent with established clinical observations. Notably, osteonecrosis of the jaw, arthralgia, pain in extremities, back pain, myalgia, and bone pain were identified as the most prevalent risk signals associated with denosumab.

## Introduction

The protein osteoprotegerin, discovered in 1997, serves as a “decoy” for receptor activators of nuclear factor kappa-B ligand (RANKL) to block bone resorption (Simonet et al., 1997). In response to this discovery, denosumab was developed, a fully human monoclonal antibody with a stronger antiresorptive activity and longer half-life which binds to RANKL to prevent RANK activation (Kendler et al., 2022). According to documented findings, the primary objective of the FREEDOM Extension study was to elucidate the long-term safety profile of denosumab, with a specific focus on the effects of prolonged inhibition of bone turnover on bone quality (Bone et al., 2017). No statistically significant variations were noted in the incidence of serious adverse events (SAEs) or adverse events (AEs) between the placebo-treated subjects and those treated with denosumab in the initial FREEDOM trial, except for SAEs related to cases of cellulitis and eczema, which were more common in the group receiving denosumab treatment (12 patients experienced cellulitis while receiving denosumab in contrast to only 1 on placebo) (Cummings et al., 2009). During the extension phase, the elderly study population exhibited a minimal incidence of adverse events, such as malignancies, cellulitis, and infections (Bone et al., 2017). In addition, previous studies have reported that multiple vertebral fractures are more likely to occur with the second dosage of denosumab (Lamy et al., 2019). Identifying and preventing osteoporosis and fragility fractures in individuals with chronic kidney disease (CKD), especially those with end-stage kidney disease (ESKD), is a complex process. The pronounced impact of denosumab-induced hypocalcemia on patients with end-stage kidney disease (ESKD) has been documented. Both high and low bone turnover, as well as lower baseline levels of 25 hydroxyvitamin D and blood calcium, are risk factors for hypocalcemia linked to denosumab usage in CKD (Gopaul et al., 2021). Therefore, it is essential to carefully select appropriate candidates for denosumab therapy, ensure adequate vitamin D and calcium supplementation, adjust calcium dialysate levels, and conduct thorough clinical monitoring of patients. FAERS, consisting of volunteered reports of adverse drug reactions (ADRs) associated with natural substances, drugs, medical devices and vaccines approved by the FDA, has been extensively employed in several studies. It serves as the benchmark approach for detecting “signals” and previously unreported ADRs (Zhang et al., 2016). There is a lack of data on FAERS analysis of RANK inhibitors, encompassing denosumab, to understand the safety of denosumab in the real world. This study aims to assess the adverse events associated with denosumab by utilizing data mining techniques on the FAERS.

## Methods

The FDA has documented AEs from 2010 to 2023, which are cataloged in the FAERS database. The web-based analysis tool, AERSMine, was created to mine the FAERS data from Q1 of 2010 to Q3 of 2023. In accordance with FDA guidelines, a deduplication process was implemented. When identical CASEIDs were encountered, the record with the most recent FDA_DT was selected. In instances where both FDA_DT and CASEID were identical, the record with the higher PRIMARYID was chosen (Chen et al., 2021). Medical Dictionary for Regulatory Activities (MedDRA) (version 25.0) system organ class (SOC) and preferred term (PT) level were employed for categorizing the AEs.

### Statistical analysis

From the first quarter of 2010 to the third quarter of 2023, FAERS reports listing “denosumab,” “Prolix,” “Kyprolia,” “Xgeva,” “Ranmark,” and “Pralia” as primary suspect drugs were analyzed after the removal of duplicate reports identified by the same ID number. Two researchers employed standardized MedDRA queries and Preferred Terms (PT) to classify AEs related to PARP inhibitors and extracted patient and drug information from the reports. We examined AEs brought on the study medications rather than illness states. To identify spontaneous signals, which were determined utilizing the case/non-case technique and indicating if there is a signal of a possible elevated risk of AE associated with the drug, a disproportionality analysis was carried out via the reporting odds ratio (ROR). Patients receiving medicine and reporting a certain AE were classified as “Cases,” with all other potential pairings being considered “non-cases.” To calculate the Reporting Risk Ratio (ROR), two-by-two contingency tables presenting counts of reported incidents for a given medicine relative to other drugs are utilized. ROR serves as a quantification of the likelihood that a particular outcome will occur in light of a specific exposure, thereby functioning as an indicator of the extent of correlation between the odds of a specific outcome and drug exposure (Rothman et al., 2004). A positive ROR signal was identified when the number of instances exceeded three, the Chi-square values surpassed four, the ROR value was greater than 2.0, and the lower limit of the 95% confidence interval (CI) was above 1.0 (Sakaeda et al., 2013). The count data were presented as frequencies (percentages), and intergroup comparisons of the count data were performed using the chi-square (χ^2^) test. Any serious AE that was found but was not mentioned in the FDA medication labeling was considered an unexpected AE. The R software was utilized for the entirety of the statistical analyses and data processing.

## Results

### Descriptive results

130611 reports on denosumab were submitted from the first quarter of 2010 through the third quarter of 2023 (i.e., the study period). Table 1 details the clinical features of denosumab-related incidents. Of all AEs, females made up a higher proportion (77.60%) than men. The majority of patients were over 65 years of age, a demographic significantly older than the median age typically observed in participants enrolled in clinical trials (Rothman et al., 2004). North America accounted for 75.03% of the reported AEs. Most of AEs were reported in 2018 (22.08%). The most commonly reported severe event was death (13.47%). Life-threatening events, disabilities, and hospitalizations occurred in 841 cases (0.57%), 2,153 cases (1.47%), and 15,952 cases (10.90%), respectively. The primary sources of reports were consumers and physicians, accounting for with 43.99% and 32.11%, respectively.

**TABLE 1:** Characteristics of reports associated with denosumab from April 2010 to October 2023.

Index	Number of events (%)
Sex	
Female (%)	113525 (77.60)
Male (%)	17086 (11.68)
Age	
<18 (%)	219 (0.15)
≥18, <45 (%)	1455 (0.99)
≥45, <65 (%)	19423 (13.28)
≥65, <75 (%)	27676 (18.92)
75≤ (%)	39201 (26.80)
Year	
2010 (%)	125 (0.09)
2011 (%)	1660 (1.13)
2012 (%)	5093 (3.48)
2013 (%)	6543 (4.47)
2014 (%)	10579 (7.23)
2015 (%)	10792 (7.38)
2016 (%)	17950 (12.27)
2017 (%)	30737 (21.01)
2018 (%)	32296 (22.08)
2019 (%)	8275 (5.66)
2020 (%)	6659 (4.55)
2021 (%)	6312 (4.31)
2022 (%)	5733 (3.92)
2023 (%)	3537 (2.42)
Reporter	
Consumer (%)	46979 (32.11)
Lawyer (%)	11 (0.01)
Other health-professional (%)	24036 (16.43)
Pharmacist (%)	9777 (6.68)
Physician (%)	64349 (43.99)
Continent of the country of occurrence	
North America (%)	109768 (75.03)
Europe (%)	19141 (13.08)
Asia (%)	6964 (4.76)
Oceania (%)	1827 (1.25)
South America (%)	1479 (1.01)
Africa (%)	52 (0.04)
Serious report	
Non-Serious (%)	75735 (51.77)
Serious (%)	70556 (48.23)
Outcome	
Life-Threatening (%)	841 (0.57)
Hospitalization - Initial or Prolonged (%)	15952 (10.90)
Disability (%)	2153 (1.47)
Death (%)	19704 (13.47)
Congenital Anomaly (%)	28 (0.02)

### Signal values related to denosumab

**FIGURE 1:**
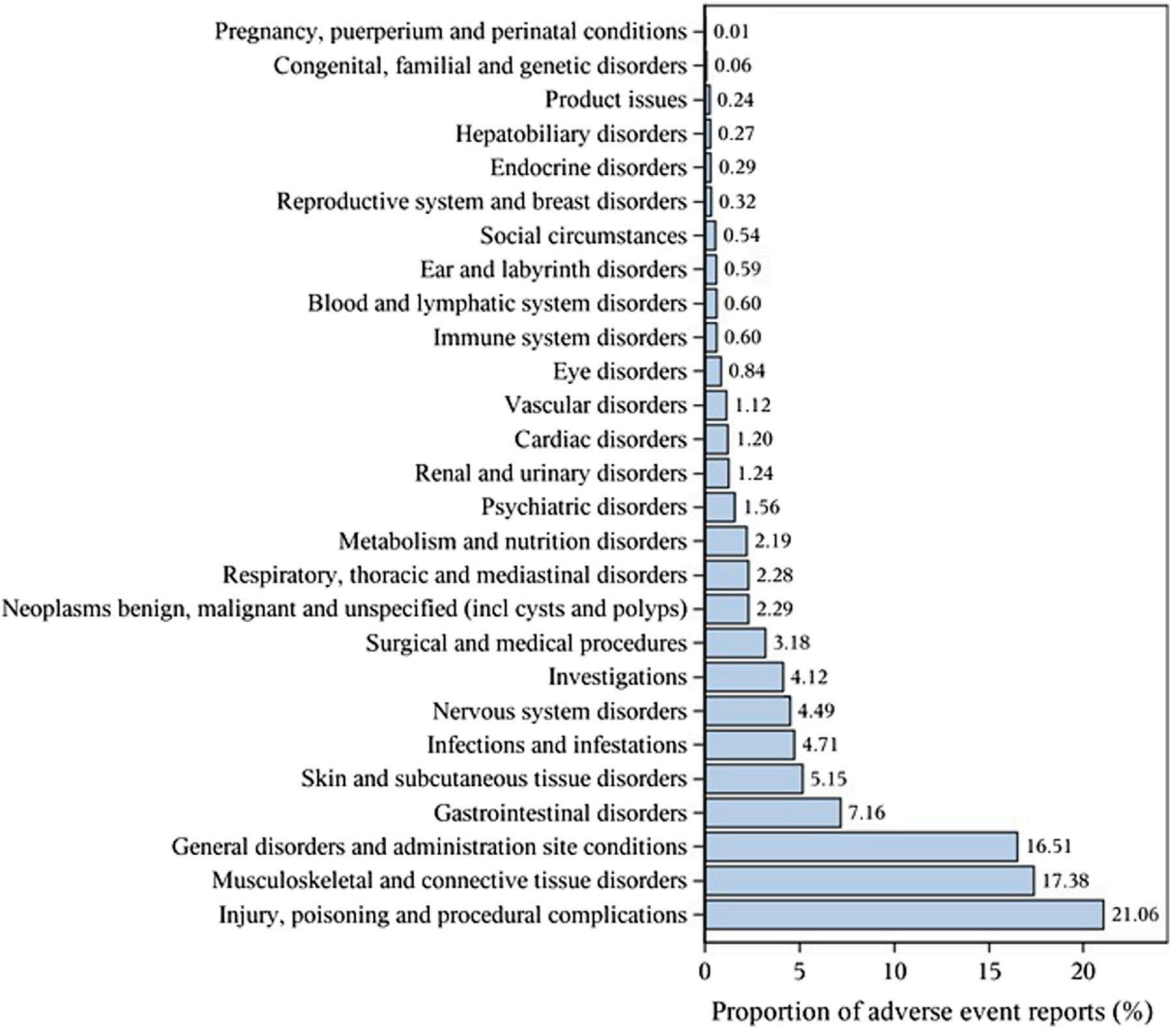
Proportion of adverse event reports (%).

The important SOCs were ‘Musculoskeletal and connective tissue disorders’ (SOC: 10,028,395),’ ‘Surgical and medical procedures (SOC: 10,042,613)’ as well as ‘Injury, poisoning and procedural complications (SOC: 10,022,117)’ (Figure 1; Table 2). Table 3 lists important PTs. Significantly, the data mining process identified several terms, including ‘Off label use (PT: 10,053,762)', ‘Death (PT: 10,011,906)', ‘Osteonecrosis of jaw (PT: 10,064,658)', ‘Arthralgia (PT: 10,003,239)', ‘Pain in extremity (PT: 10,033,425)', ‘Back pain (PT: 10,003,988)', ‘Myalgia (PT: 10,028,411)', ‘Bone pain (PT: 10,006,002)', ‘Tooth disorder (PT: 10,044,034)', and ‘Hypocalcaemia (PT: 10,020,947)'. Events of osteonecrosis of jaw (PT: 10,064,658) and hypocalcaemia (PT: 10,020,947) were reported in patients with denosumab treatment, as noted in the denosumab labeling. In our analysis, joint disorders such as osteoarthritis (PT: 10,057,178), arthritis (PT: 10,003,284), and arthralgia (PT: 10,023,226) were identified, which were associated with clinical trial outcomes.

**TABLE 2:** Signal strength of AEs of denosumab at the SOC level in FAERS database.

SOC	Cases	ROR	95%CI lower	95%CI upper	Chi-square
Injury, poisoning and procedural complications	63717	**2.298115725**	**2.277995715**	**2.318413442**	36397.88275
Musculoskeletal and connective tissue disorders	52575	**3.835615349**	**3.799352711**	**3.872224092**	89066.44648
General disorders and administration site conditions	49939	0.912789786	0.90404174	0.921622483	345.1542041
Gastrointestinal disorders	21673	0.828185658	0.816787179	0.839743207	712.9283675
Skin and subcutaneous tissue disorders	15584	0.936695478	0.921666999	0.951969008	62.82528049
Infections and infestations	14251	0.889402418	0.874522211	0.904535815	185.5943595
Nervous system disorders	13593	0.511599235	0.502857167	0.520493282	6028.814339
Investigations	12467	0.689696739	0.677412306	0.702203943	1660.43858
Surgical and medical procedures	9632	**2.484979224**	**2.43464043**	**2.536358826**	8137.974517
Neoplasms benign, malignant and unspecified (incl cysts and polyps)	6915	0.797661827	0.778815072	0.81696466	344.862688
Respiratory, thoracic and mediastinal disorders	6913	0.475757095	0.464526409	0.487259301	3888.276227
Metabolism and nutrition disorders	6640	1.043628439	1.018462134	1.069416605	11.75923122
Psychiatric disorders	4705	0.267195086	0.259602272	0.275009973	9288.157057
Renal and urinary disorders	3754	0.633858437	0.613735213	0.654641463	780.5955163
Cardiac disorders	3617	0.476174576	0.460789955	0.492072852	2052.264271
Vascular disorders	3403	0.529451373	0.511827808	0.547681763	1401.796782
Eye disorders	2540	0.421416932	0.405252199	0.438226447	1994.653087
Immune system disorders	1827	0.534550228	0.51047671	0.559759028	733.2028226
Blood and lymphatic system disorders	1823	0.364349376	0.347932777	0.381540563	2004.172329
Ear and labyrinth disorders	1789	1.360526864	1.298455614	1.425565362	168.3103294
Social circumstances	1622	1.195178865	1.138026506	1.255201449	50.9974149
Reproductive system and breast disorders	958	0.348091032	0.32667341	0.370912854	1163.032048
Endocrine disorders	875	1.161653979	1.086781483	1.241684724	19.46897088
Hepatobiliary disorders	830	0.313851178	0.293159658	0.336003127	1238.865644
Product issues	724	0.143907023	0.133780397	0.154800193	3674.286997
Congenital, familial and genetic disorders	170	0.185821614	0.159863741	0.215994396	605.3077496
Pregnancy, puerperium and perinatal conditions	22	0.017359173	0.011429606	0.026364943	1223.450674

Abbreviations: SOC, system organ class; CI, confidence interval; ROR, reporting odds ratio; AEs, Adverse effects; FAERS, food and drug administration adverse event reporting system.

Used to emphasize the important SOCs.

**TABLE 3:** Signal strength of AEs of denosumab at the Preferred Terms level in FAERS database.

PT	Cases	ROR	95%CI lower	95%CI upper	Chi-square
Off label use	33435	9.763330018	9.650003114	9.877987802	220555.3608
Death	17071	4.10378186	4.040079703	4.168488444	36814.48507
Osteonecrosis of jaw	7418	74.968043	72.88337577	77.11233752	349697.1632
Arthralgia	5995	3.029451587	2.952201681	3.108722881	7826.470245
Pain in extremity	5116	3.414259489	3.320129911	3.511057752	8389.338553
Back pain	4977	4.358136495	4.235995922	4.483798865	12298.47791
Myalgia	3213	4.036789875	3.897004424	4.181589426	7065.180406
Bone pain	3066	11.09721631	10.69513468	11.51441412	25897.92117
Tooth disorder	2697	26.71521661	25.63595466	27.83991501	55846.25009
Hypocalcaemia	2552	34.7639592	33.28870079	36.30459678	66893.16525
Pain in jaw	2248	17.07837657	16.34545446	17.84416254	30202.78721
Hospitalisation	2094	2.874170207	2.752083022	3.001673392	2491.695531
Spinal fracture	2046	24.06421346	22.96065728	25.22080977	38501.84212
Product storage error	1856	4.434160357	4.233413947	4.644426064	4759.691245
Musculoskeletal pain	1440	4.981250533	4.725728641	5.250588589	4407.783151
Tooth extraction	1356	30.27125975	28.54646334	32.10026951	31570.54231
Mobility decreased	1261	3.508591274	3.31757947	3.710600708	2198.692107
Fracture	1172	12.56602017	11.83650254	13.34050006	11430.03623
Accidental exposure to product	1124	2.723386887	2.567041878	2.889254047	1198.552889
Adverse drug reaction	1121	2.612825544	2.462688476	2.772115673	1092.034675
Adverse event	1117	2.468754418	2.326718082	2.619461473	956.0330819
Femur fracture	1112	6.694556015	6.303207826	7.110201895	5127.989864
Hip fracture	1031	6.729659433	6.321569958	7.164093159	4788.262143
Toothache	982	11.68834284	10.95125637	12.47503973	8847.600597
Circumstance or information capable of leading to medication error	970	6.95767613	6.52283385	7.421507007	4704.816024
Tooth fracture	915	17.33862099	16.1876796	18.57139413	12533.35046
Bone density decreased	848	4.689667955	4.379223862	5.022119495	2377.186067
Blood calcium decreased	838	17.403859	16.19840017	18.69902614	11525.06468
Jaw disorder	829	40.52088943	37.51191726	43.7712226	24858.02546
Impaired healing	791	5.446015882	5.072306141	5.847259247	2758.965257
Cystitis	790	4.785031116	4.457180204	5.136997325	2283.063825
Neoplasm malignant	705	2.074805561	1.925973747	2.235138523	386.0356747
Incorrect route of product administration	686	2.449071753	2.270852383	2.641278004	576.9947529
Atypical femur fracture	680	57.82946197	52.90036111	63.21784202	27014.74723
Groin pain	679	16.55978149	15.29399714	17.93032654	8880.985841
Spinal compression fracture	660	15.80021586	14.57873148	17.12404277	8225.164753
Neck pain	652	2.360994071	2.184990752	2.551174645	502.1629882
Rash pruritic	647	2.556359308	2.364964243	2.763243855	601.046642
Osteomyelitis	609	6.99354287	6.446735705	7.586729798	2976.878479
Bone density abnormal	607	32.37937563	29.64761026	35.36284906	15032.63939
Bone disorder	602	6.793951152	6.260139048	7.373282271	2834.543224
Terminal state	594	17.88065359	16.41776663	19.47388947	8401.624884
Arthropathy	582	2.10798295	1.94218573	2.287933666	333.3911754
Tooth loss	575	5.095734443	4.68867947	5.538128525	1824.5376
Eczema	553	3.691266691	3.392208134	4.016690382	1055.950269
Metastases to bone	547	7.357497453	6.751204542	8.018238587	2853.251707
Osteoarthritis	545	2.632461142	2.418432947	2.865430557	540.8100751
Surgery	528	2.047720387	1.87901546	2.231572263	278.5752368
Cellulitis	528	2.067072936	1.8967628	2.252675203	286.2180999
Dental caries	522	9.935002746	9.091010128	10.85735008	3916.360302
Blood cholesterol increased	518	2.497531061	2.289572637	2.724378034	456.3879751
Dental care	509	76.99861828	69.13396163	85.75795567	24810.11002
Occupational exposure to product	483	15.75270598	14.33916338	17.30559442	6003.737474
Foot fracture	473	4.782941814	4.363940529	5.242173271	1367.445906
Intercepted product administration error	465	22.70918444	20.59163048	25.04449846	8319.26406
Osteonecrosis	462	3.500558442	3.191720206	3.83928058	804.3583374
Hypercalcaemia	459	8.222704716	7.483958217	9.034373374	2750.082291
Tooth infection	453	6.928238942	6.304427598	7.613775255	2188.784822
Pathological fracture	444	19.72245933	17.85869573	21.78072845	6927.773971
Lumbar vertebral fracture	444	22.53318896	20.38648118	24.90594626	7885.142655
Rib fracture	442	4.304845119	3.915948685	4.732363212	1087.168978
Blood parathyroid hormone increased	441	36.43883465	32.82078284	40.45572823	12104.468
Body height decreased	429	7.220253077	6.552474831	7.956086188	2185.688
Thoracic vertebral fracture	425	36.26320611	32.60070325	40.33717025	11617.73354
Bone loss	406	4.533366112	4.106626711	5.004450064	1082.410747
Tooth abscess	401	7.918844419	7.160926108	8.756981429	2294.283734
Ear pain	401	4.275742102	3.87121226	4.722544074	975.9446277
Wrist fracture	392	6.580508082	5.946537377	7.282067508	1771.491818
Loose tooth	369	25.22507826	22.58173914	28.17783738	7292.308614
Adverse reaction	364	5.206338687	4.689079279	5.780657759	1192.27321
Dental implantation	358	60.22118003	53.22587884	68.13585052	14668.37783
Rebound effect	354	8.232746884	7.396004688	9.164153367	2124.844168
Pelvic fracture	352	8.555116776	7.682400362	9.526973291	2213.818882
Upper limb fracture	347	3.22188823	2.896531467	3.583791127	519.421664
Blood calcium increased	340	8.228284299	7.375895089	9.179179162	2039.548062
Multiple fractures	335	3.428973067	3.07665532	3.821635858	562.3246446
Diverticulitis	320	2.365988906	2.118420348	2.642489489	248.0088899
Oral pain	314	2.750581328	2.459824814	3.07570588	342.9022015
Sciatica	311	4.4342269	3.960817009	4.9642203	801.5554077
Lower limb fracture	303	3.421814554	3.053170259	3.834969506	506.7515354
Musculoskeletal discomfort	299	3.427621607	3.05602372	3.844404022	501.5981269
Gingival pain	299	8.052963097	7.16707196	9.048355452	1747.070952
Exposed bone in jaw	288	22.90135133	20.22202875	25.93567141	5196.814323
Gingival disorder	272	15.11664048	13.34040217	17.12938008	3240.966041
Knee arthroplasty	271	3.014523372	2.672681197	3.400087961	357.0041814
Musculoskeletal disorder	271	2.541102649	2.253383887	2.8655582	248.653254
Compression fracture	265	13.04867432	11.50608994	14.79806802	2700.016937
Hip arthroplasty	264	4.038568821	3.573415078	4.564271927	586.5117965
Hypophosphataemia	264	7.829931081	6.917286278	8.862987342	1490.108607
Endodontic procedure	263	23.96749034	21.03280273	27.31165222	4955.422334
Dermatitis	262	2.772349743	2.453189003	3.133033407	290.9788978
Vitamin D decreased	257	5.561967682	4.91008387	6.300398388	924.9499208
Spinal pain	253	3.987573081	3.519102736	4.518407182	550.5245401
Temporomandibular joint syndrome	250	17.37313812	15.23541092	19.81081637	3438.074382
Stress fracture	250	8.571935149	7.54459593	9.739166003	1576.551756
Gingivitis	239	8.225939122	7.220154987	9.371831292	1433.63602
Breast cancer metastatic	228	5.309706412	4.652025997	6.060366429	768.5018632
Dental operation	226	25.36290164	22.01680867	29.21753053	4490.846164
Exostosis of jaw	226	93.1450675	78.78136511	110.1276119	12480.66752
Musculoskeletal chest pain	219	2.874597864	2.514564024	3.286181144	262.2706937
Back disorder	218	2.619229664	2.290746113	2.994816403	214.1541017
Ankle fracture	208	2.629239086	2.292185945	3.01585401	206.069567
Hospice care	207	4.215725618	3.671344782	4.84082633	492.9200436
Exostosis	201	4.705728399	4.088741535	5.415817943	567.5527578
Metastases to liver	196	2.328964925	2.022321509	2.682104501	146.1644819
Vitamin D deficiency	195	3.787192814	3.285044087	4.366099521	389.449295
Investigation	194	9.653106	8.346621382	11.16409277	1408.995217
Sleep disorder due to a general medical condition	192	4.142472547	3.588623748	4.781799376	444.5714584
Spinal deformity	190	23.24859397	19.94376157	27.10106214	3478.896819
Walking aid user	186	6.338284994	5.472352804	7.341240249	800.3774812
Hyperlipidaemia	180	3.353500998	2.892650667	3.887772923	290.3387456
Spinal operation	179	3.446847434	2.971804026	3.997826617	303.43955
Blood calcium abnormal	176	23.56539936	20.09198678	27.63927993	3264.343857
Femoral neck fracture	171	7.013661859	6.015331309	8.177679689	840.12855
Oral disorder	169	4.539488812	3.894772816	5.290927006	451.8382511
Bedridden	167	2.562081672	2.198465744	2.985837969	156.1707414
Bone giant cell tumour	167	570.1854986	406.5099075	799.7628024	19057.80329
Immune system disorder	164	2.513072515	2.153466418	2.932729023	146.7496548
Spinal disorder	160	3.349373426	2.863318931	3.917936709	257.5152855
Hyperaesthesia teeth	160	11.48844557	9.779249012	13.49637191	1417.687845
Humerus fracture	157	6.622919713	5.643471871	7.772354771	716.0432372
Gingival swelling	157	7.454139361	6.348954195	8.751708062	833.5746222
Paraesthesia oral	156	2.30943864	1.971470185	2.705344912	113.9265788
Device failure	147	2.09395134	1.779227309	2.464346287	82.76349422
Osteitis	147	12.83655308	10.84299995	15.19663338	1471.880385
Gingival bleeding	138	2.189132486	1.850307664	2.590002267	87.75833028
Mastication disorder	133	9.989118668	8.378641384	11.90914937	1005.354267
Gingival recession	132	15.78490175	13.18759671	18.89374757	1645.969472
Atypical fracture	128	64.17035939	52.09541212	79.04410114	5496.489055
Abscess jaw	123	26.76081379	22.07378268	32.44306448	2569.379177
Osteoporotic fracture	121	14.71441852	12.20312178	17.74251836	1402.151427
Scoliosis	120	3.674386644	3.06531345	4.404481769	227.6578451
Hyperparathyroidism	120	12.97496972	10.76332411	15.64106381	1215.703845
Bursitis	119	2.357525514	1.966849386	2.825801809	91.48128931
Tetany	119	15.62405869	12.93023641	18.87909874	1468.242186
Blood phosphorus decreased	117	7.030223446	5.839191944	8.464191994	576.6684022
Trismus	115	4.873451978	4.0467602	5.869024357	342.2752528
Metastases to lung	114	2.068840049	1.719550229	2.48908062	62.03221123
Jaw fracture	114	10.71748859	8.859893522	12.96455328	934.2178279
Joint noise	113	5.487877187	4.547739733	6.622365788	399.282751
Dental restoration failure	112	41.81704847	33.87875711	51.61539832	3453.326811
Periodontitis	112	11.08933191	9.149676831	13.44017766	953.9412749
Skin infection	112	2.064687404	1.713285664	2.488163045	60.59503982
Artificial crown procedure	111	59.38195459	47.59511209	74.08778708	4503.633715
Spinal stenosis	111	2.497586562	2.070193805	3.013214812	97.93235078
Jaw operation	109	37.29704242	30.20902968	46.04813158	3054.353147
Oral infection	109	5.734543014	4.735194706	6.944800714	409.5386405
Cataract operation	107	3.877493839	3.19993336	4.698522368	222.3914572
Oral surgery	105	10.94000338	8.970609715	13.34175466	880.7618341
Tibia fracture	105	4.446897254	3.661794409	5.400329176	272.0020589
Facial pain	105	2.416788732	1.992767675	2.93103298	85.73334804
Immobile	104	5.382508983	4.425367903	6.546665403	357.5516159
Radius fracture	104	8.583710468	7.042548722	10.46213357	657.2330626
Nerve compression	101	2.174204	1.786269422	2.646388599	63.07046822
Muscle disorder	101	2.555755757	2.099202097	3.111604879	93.94328587
Metastasis	100	2.975968294	2.441257221	3.627797682	128.4887717

Abbreviations: SOC, system organ class; CI, confidence interval; ROR, reporting odds ratio; AEs, Adverse effects; FAERS, food and drug administration adverse event reporting system.

### Onset time of events

The onset times of AEs associated with denosumab were extracted from the database. After excluding false reports (n = 130,301, 89.07%), a total of 15,990 AEs with reported onset times were analyzed. The median onset time was 110 days, with an interquartile range (IQR) of 5–443 days. As illustrated in Table 1, the data suggest that the onset of denosumab-related AEs can span over a year. However, the majority of cases (n = 6,137, 4.2%) occurred within the first month following denosumab initiation. The incidence rates of AEs observed at 2 months (n = 886, 0.61%), 3 months (n = 632, 0.43%), 4 months (n = 516, 0.35%), 5 months (n = 453, 0.31%), and 6 months (n = 481, 0.33%) were comparable, indicating that AEs may occur at any point within the first year of treatment. Additionally, the data demonstrated that AEs occurred after 1 year of denosumab treatment at a rate of 3.26% (n = 4767).

## Discussion

Denosumab is one of the human monoclonal antibodies targeting RANKL, a potent inhibitor of osteoclast activity and differentiation and is used in the treatment of osteoporosis and bone metastasis (Cummings et al., 2009; Delmas, 2008). Denosumab, as the first biologic agent utilized in the management of osteoporosis, has exhibited significant anti-resorptive properties and effectiveness in fracture prevention (Ferrari and Langdahl, 2023). Because the systematic review of AEs for denosumab is lacking, this work was conducted to investigate the AEs in patients following denosumab, and to provide reference for clinical safety applications. This study utilizes the FAERS pharmaceutical database to elucidate the various AEs linked to the use of denosumab and is one of the most extensive collections of such cases in history.

About 48.23% of patients receiving denosumab treatment experienced significant intolerances (some of which were even fatal, accounting for 13.47%), which indicates that greater emphasis should be placed on addressing the safety concerns associated with denosumab, in addition to fatal complications, it can be seen that the drug has many different non-fatal AEs, which also plague patients. Furthermore, the study demonstrated that the most prevalent adverse events (AEs) in patients receiving denosumab included osteonecrosis of the jaw, arthralgia, pain in the extremities, back pain, myalgia, bone pain, tooth disorders, and hypocalcemia. Previous investigations have reported that osteonecrosis of the jaw is a common complication of denosumab therapy (Ahdi et al., 2023). Furthermore, the study indicated that drug-associated ONJ represents a significant adverse reaction observed in certain individuals administered commonly used medications for cancer and osteoporosis treatment, such as denosumab and anti-angiogenic agents. This condition is characterized by progressive bone destruction of the mandible or maxilla (Beth-Tasdogan et al., 2022). Our analysis also revealed that ONJ is top-ranked AE. A variety of antiremodeling or antiresorptive drugs, like monoclonal antibodies, bisphosphonates, hormonal replacement therapy, and are commonly administered to a multitude of patients. ONJ is a consequence of decreased bone turnover resulting from the administration of antiresorptive drugs (Uyanne et al., 2014). This reduction in bone turnover is ascribed to the antiresorptive characteristics of both denosumab and bisphosphonates since they impede the bone remodeling process by suppressing osteoclast activity and inducing cellular apoptosis (Zhang et al., 2016). One systematic review and meta-analysis (Boquete-Castro et al., 2016) comprising 7 randomized clinical trials in individuals with cancer treated by denosumab showed that the adverse effects of denosumab displayed that ONJ has an overall incidence of 1.7% [95% CI: 0.9%-3.1%]. Moreover, the study also revealed that pain in extremity, back pain, myalgia, and bone pain are common AEs for patients following denosumab drug use. Similar results have been reported by Vasiliki, et al. (Chatziravdeli et al., 2022). Yumie et al. demonstrated that the major AEs observed in more than 0.5% of patients were arthralgia (0.7%), dizziness (0.7%), myalgia (0.6%), and back pain (0.6%). Tomonori and his colleagues further demonstrated that 1.4% of patients experienced blindness, limb numbness, and diarrhea, while 4.3% of patients sustained new fractures (Kobayakawa et al., 2021). Furthermore, hypocalcemia was found to occur in 0.3% of postmenopausal Korean women with osteoporosis (Rhee et al., 2022). Huang et, al (Huang et al., 2020) reported that in both groups (zoledronic acid, denosumab), 194 patients who received a minimum of one dosage of the study medication had at least one treatment-emergent AE, and 18.6% of patients experienced bone pain in multiple myeloma. Furthermore, the study findings indicate that the prevailing AEs reported in both zoledronic acid and denosumab groups were pyrexia (38.2%, 41.3%), nausea (42.2%, 46.7%), and diarrhea (51.0%, 51.1%). However, our analysis did not identify these symptoms as statistically significant signals, emphasizing the need to enhance management strategies for these AEs (Huang et al., 2020). However, one study reported that denosumab has been displayed to reduce bone pain in individuals with multiple myeloma, breast as well as prostate cancer by avoiding skeletal-associated events, and the findings suggest that denosumab may confer benefits in pain prevention through the delay of bone pain onset, instead of producing direct analgesia (Porta-Sales et al., 2017). Furthermore, research has demonstrated that individuals with a history of mental illness experience recurrent episodes of acute respiratory complications and depressive relapse, which are often accompanied by heightened anxiety and psychomotor inhibition. These occurrences were observed in patients who received sequential administrations of denosumab, without any underlying calculus/phase imbalance, which is considered uncommon adverse events (Oteo-Álvaro et al., 2023), and based on our results, nervous system disorders were not significant signals.

### Limitation

There were several limitations in the study. FAERS database, a spontaneous reporting system (SRS), the data mining method applied in this work did not improve it because of its inherent limitations. SRS utilized for signal detection relies on data from both clinical trials and post-marketing reports. Nevertheless, these systems are limited by the registration of only observed adverse events, leading to potential underreporting and reporting bias (Noguchi et al., 2021). The voluntary nature of adverse drug reaction (ADR) reporting within the FAERS system presents notable challenges, including under-reporting together with possible reporting biases like uneven information quality, false reporting, and inaccuracy. Furthermore, other biases including the weber effect, notoriety effects, masking effect or cloaking effect might be caused due to SRS which might have an impact on the results, thus, it is crucial to comprehend the range of biases present in adverse event reporting in order to accurately interpret data (Noguchi et al., 2021). Furthermore, although data mining techniques facilitated the identification of adverse reaction signals associated with denosumab, this alone does not establish a causal relationship. It is imperative to validate these findings through prospective studies. Future large-scale, population-based prospective studies are necessary to accurately determine the incidence of denosumab-related potential adverse events and to comprehensively elucidate the underlying biological mechanisms and risk factors, thereby enhancing risk management strategies. Furthermore, the utilization or exposure to multiple medications presents a challenge in discerning the specific etiology of adverse events, as well as determining the clinical indication for these pharmaceutical agents**.**

## Conclusion

The current investigation, utilizing the FAERS database, has indicated potential safety concerns regarding the utilization of denosumab, specifically in relation to an elevated likelihood of experiencing osteonecrosis of the jaw, arthralgia, pain in extremities, back pain, myalgia, and bone pain events. Additionally, unforeseen adverse events such as nervous disorder may also manifest. Consequently, it is advised that vigilant monitoring and identification of these adverse events be implemented across all populations. To substantiate these findings and gain a more comprehensive understanding of denosumab’s safety profile, further research in the form of cohort studies and long-term clinical investigations is warranted.

## Data Availability

The raw data supporting the conclusions of this article will be made available by the authors, without undue reservation.

## References

[B1] Ahdi H. S. Wichelmann T. A. Pandravada S. Ehrenpreis E. D. (2023). Medication-induced osteonecrosis of the jaw: a review of cases from the Food and Arug administration adverse event reporting system (FAERS). BMC Pharmacol. Toxicol. 24 (1), 15. 10.1186/s40360-023-00657-y 36879299 PMC9987072

[B2] Beth-Tasdogan N. H. Mayer B. Hussein H. Zolk O. (2022). Interventions for managing medication-related osteonecrosis of the jaw. Cochrane Database Syst. Rev. 7 (7), Cd012432. 10.1002/14651858.CD012432.pub2 35866376 PMC9309005

[B3] Bone H. G. Wagman R. B. Brandi M. L. Brown J. P. Chapurlat R. Cummings S. R. (2017). 10 years of denosumab treatment in postmenopausal women with osteoporosis: results from the phase 3 randomised FREEDOM trial and open-label extension. Lancet Diabetes Endocrinol. 5 (7), 513–523. 10.1016/S2213-8587(17)30138-9 28546097

[B4] Boquete-Castro A. Gómez-Moreno G. Calvo-Guirado J. L. Aguilar-Salvatierra A. Delgado-Ruiz R. A. (2016). Denosumab and osteonecrosis of the jaw. A systematic analysis of events reported in clinical trials. Clin. Oral Implants Res. 27 (3), 367–375. 10.1111/clr.12556 25639776

[B5] Chatziravdeli V. Katsaras G. N. Katsaras D. Doxani C. Stefanidis I. Zintzaras E. (2022). A systematic review and meta-analysis of interventional studies of bisphosphonates and denosumab in multiple myeloma and future perspectives. J. Musculoskelet. Neuronal Interact. 22 (4), 596–621. 36458395 PMC9716295

[B6] Chen C. Chen T. Liang J. Guo X. Xu J. Zheng Y. (2021). Cardiotoxicity induced by immune checkpoint inhibitors: a pharmacovigilance study from 2014 to 2019 based on FAERS. Front. Pharmacol. 12, 616505. 10.3389/fphar.2021.616505 33643048 PMC7907652

[B7] Cummings S. R. San Martin J. McClung M. R. Siris E. S. Eastell R. Reid I. R. (2009). Denosumab for prevention of fractures in postmenopausal women with osteoporosis. N. Engl. J. Med. 361 (8), 756–765. 10.1056/NEJMoa0809493 19671655

[B8] Delmas P. D. (2008). Clinical potential of RANKL inhibition for the management of postmenopausal osteoporosis and other metabolic bone diseases. J. Clin. Densitom. 11 (2), 325–338. 10.1016/j.jocd.2008.02.002 18375161

[B9] Ferrari S. Langdahl B. (2023). Mechanisms underlying the long-term and withdrawal effects of denosumab therapy on bone. Nat. Rev. Rheumatol. 19 (5), 307–317. 10.1038/s41584-023-00935-3 37024711

[B10] Gopaul A. Kanagalingam T. Thain J. Khan T. Cowan A. Sultan N. (2021). Denosumab in chronic kidney disease: a narrative review of treatment efficacy and safety. Arch. Osteoporos. 16 (1), 116. 10.1007/s11657-021-00971-0 34319515

[B11] Huang S. Y. Yoon S. S. Shimizu K. Chng W. J. Chang C. S. Wong R. S. M. (2020). Denosumab versus zoledronic acid in bone disease treatment of newly diagnosed multiple myeloma: an international, double-blind, randomized controlled phase 3 study-asian subgroup analysis. Adv. Ther. 37 (7), 3404–3416. 10.1007/s12325-020-01395-x 32524500 PMC7467415

[B12] Kendler D. L. Cosman F. Stad R. K. Ferrari S. (2022). Denosumab in the treatment of osteoporosis: 10 Years later: a narrative review. Adv. Ther. 39 (1), 58–74. 10.1007/s12325-021-01936-y 34762286 PMC8799550

[B13] Kobayakawa T. Miyazaki A. Saito M. Suzuki T. Takahashi J. Nakamura Y. (2021). Denosumab versus romosozumab for postmenopausal osteoporosis treatment. Sci. Rep. 11 (1), 11801. 10.1038/s41598-021-91248-6 34083636 PMC8175428

[B14] Lamy O. Stoll D. Aubry-Rozier B. Rodriguez E. G. (2019). Stopping denosumab. Curr. Osteoporos. Rep. 17 (1), 8–15. 10.1007/s11914-019-00502-4 30659428

[B15] Noguchi Y. Tachi T. Teramachi H. (2021). Detection algorithms and attentive points of safety signal using spontaneous reporting systems as a clinical data source. Brief. Bioinform 22 (6), bbab347. 10.1093/bib/bbab347 34453158

[B16] Oteo-Álvaro Á. García C. G. Sánchez A. I. Santamaria C. A. de Diego-Adeliño J. (2023). Neuropsychiatric adverse reactions in patients treated with denosumab: two case reports and a review of data from the FDA Adverse Event Reporting System (FAERS). Osteoporos. Int. 34 (10), 1799–1804. 10.1007/s00198-023-06838-z 37405407

[B17] Porta-Sales J. Garzón-Rodríguez C. Llorens-Torromé S. Brunelli C. Pigni A. Caraceni A. (2017). Evidence on the analgesic role of bisphosphonates and denosumab in the treatment of pain due to bone metastases: a systematic review within the European Association for Palliative Care guidelines project. Palliat. Med. 31 (1), 5–25. 10.1177/0269216316639793 27006430

[B18] Rhee Y. Chang D. G. Ha J. Kim S. Lee Y. Jo E. (2022). Real-world safety and effectiveness of denosumab in patients with osteoporosis: a prospective, observational study in South Korea. Endocrinol. Metab. Seoul. 37 (3), 497–505. 10.3803/EnM.2022.1427 35654577 PMC9262695

[B19] Rothman K. J. Lanes S. Sacks S. T. (2004). The reporting odds ratio and its advantages over the proportional reporting ratio. Pharmacoepidemiol Drug Saf. 13 (8), 519–523. 10.1002/pds.1001 15317031

[B20] Sakaeda T. Tamon A. Kadoyama K. Okuno Y. (2013). Data mining of the public version of the FDA adverse event reporting system. Int. J. Med. Sci. 10 (7), 796–803. 10.7150/ijms.6048 23794943 PMC3689877

[B21] Simonet W. S. Lacey D. L. Dunstan C. R. Kelley M. Chang M. S. Lüthy R. (1997). Osteoprotegerin: a novel secreted protein involved in the regulation of bone density. Cell 89 (2), 309–319. 10.1016/s0092-8674(00)80209-3 9108485

[B22] Uyanne J. Calhoun C. C. Le A. D. (2014). Antiresorptive drug-related osteonecrosis of the jaw. Dent. Clin. North Am. 58 (2), 369–384. 10.1016/j.cden.2013.12.006 24655528

[B23] Zhang X. Hamadeh I. S. Song S. Katz J. Moreb J. S. Langaee T. Y. (2016). Osteonecrosis of the jaw in the United States Food and drug administration's adverse event reporting system (FAERS). J. Bone Min. Res. 31 (2), 336–340. 10.1002/jbmr.2693 26288087

